# *In Vitro* Performance of Dutasteride-Nanostructured Lipid Carriers Coated with Lauric Acid-Chitosan Oligomer for Dermal Delivery

**DOI:** 10.3390/pharmaceutics12100994

**Published:** 2020-10-20

**Authors:** Norhayati Mohamed Noor, Azila Abdul-Aziz, Khalid Sheikh, Satyanarayana Somavarapu, Kevin M. G. Taylor

**Affiliations:** 1 Department of Pharmaceutics, UCL School of Pharmacy, 29–39 Brunswick Square, London WC1N 1AX, UK; k.sheikh@ucl.ac.uk (K.S.); s.somavarapu@ucl.ac.uk (S.S.); 2 Cosmeceutical & Fragrance Laboratory, Institute of Bioproduct Development (N22), Universiti Teknologi Malaysia, UTM Johor Bahru 81310, Johor, Malaysia; azila@ibd.utm.my; 3 School of Chemical and Energy Engineering, Faculty of Engineering, Universiti Teknologi Malaysia, UTM Johor Bahru 81310, Johor, Malaysia

**Keywords:** androgenic alopecia, chitosan, dermal delivery, dutasteride, lauric acid, nanostructured lipid carriers, human hair follicle dermal papilla cells, reconstructed human epidermis

## Abstract

Dutasteride, licensed as an oral medicine for the treatment of benign prostatic hypoplasia, has been investigated as a treatment for androgenic alopecia. In this study, the potential for dustasteride to be delivered topically in order to reduce systemic exposure, irritation of the skin, and also cytotoxicity was explored. Chitosan oligomer (CSO) was successfully synthesised with lauric acid as a coating for a dutasteride-loaded nanostructured lipid carriers (DST-NLCs) system. DST-NLCs were prepared using a combination of melt-dispersion and ultrasonication. These negatively charged NLCs (−18.0 mV) had a mean particle size of ~184 nm, which was not significantly increased (*p >* 0.05) when coated with lauric acid-chitosan oligomer (CSO-LA), whilst the surface charge changed to positive (+24.8 mV). The entrapment efficiency of DST-NLCs was 97%, and coated and uncoated preparations were physically stable for up to 180 days at 4–8 °C. The drug release was slower from DST-NLCs coated with CSO-LA than from uncoated NLCs, with no detectable drug permeation through full-thickness pig ear skin from either preparation. Considering the cytotoxicity, the IC_50_ values for the DST-NLCs, coated and uncoated with CSO-LA were greater than for dutasteride alone (*p <* 0.05). DST-NLCs and empty NLCs coated with CSO-LA at 25 µM increased the cell proliferation compared to the control, and no skin irritation was observed when the DST-NLC formulations were tested using EpiDerm™. The cell and skin uptake studies of coated and uncoated NLCs incorporating the fluorescent marker Coumarin-6 showed the time-dependent uptake of Coumarin-6. Overall, the findings suggest that DST-NLCs coated with CSO-LA represent a promising formulation strategy for dutasteride delivery for the treatment of androgenic alopecia, with a reduced cytotoxicity compared to that of the drug alone and lower irritancy than an ethanolic solution of dutasteride.

## 1. Introduction

Male pattern hair loss (MPHL), also known as androgenic alopecia (AGA), is the most common type of hair loss in men, with a prevalence of ~20% at age 20 to 30, and of 30% to 50% of men by age 50 [1,2,3]. AGA has a close genetic component, with heredity accounting for approximately 80% of predisposition, although normal levels of androgens are sufficient to cause hair loss in genetically susceptible individuals [3]. Androgens are capable of regulating and stimulating hair growth. In affected hair-loss individuals, they reverse the transformation of large, deep follicles producing long, often heavily pigmented terminal scalp hairs to miniaturised vellus follicles forming tiny, almost invisible hairs [4]. Testosterone, a key androgen, is converted to its potent form, dihydrotestosterone (DHT), by the 5α-reductase enzyme, which appears to have a higher activity on the balding scalp compared to the non-balding scalp [5]. In hair-loss patients, 5α-reductase acts as a catalyst that converts testosterone to DHT, which makes the hair follicle miniaturise and shed hair [6]. The psycho-social consequences of baldness can be significant, especially for younger men [7]. Currently, there are two approved treatments for hair loss—minoxidil, which is the only topical-based product, and finasteride (type II 5α-reductase inhibitor), which is available as oral tablets. There is an unfulfilled need for alopecia treatments which provide satisfactory, long-term clinical results [8]. A sustained release delivery system could potentially be used to prolong the duration of treatment.

Dutasteride is approved by the United States Food and Drug Administration (FDA) as an oral treatment for benign prostatic hyperplasia [9]. It is a dual type I and type II 5α-reductase inhibitor which has been shown to decrease serum DHT by more than 90% compared to finasteride, which decreased serum DHT by 70% [10]. Olsen et al. [6] in a randomised placebo-controlled trial found that the dutasteride-group (2.5 mg) increased the scalp hair count more than the finasteride-group (5 mg) in men between the ages of 21 and 45 years with MPHL. Due to the systemic effects of lowered DHT levels, patients receiving oral dutasteride have reported diminished sexual desire, increased depression, and ejaculation disorders [11]. Consequently, a topical formulation of dutasteride for local application to the balding area, thereby minimizing systemic adverse effects, would be an appropriate drug-delivery strategy.

Dutasteride is poorly water soluble (0.038 ng/mL; Log *p* = 5.09 [12]). However, an alcohol-based vehicle is not recommended for drug delivery, as this may cause contact dermatitis and skin irritation [13]. Moreover, with a propylene glycol-based minoxidil preparation (minoxidil is widely used for the treatment of hair loss), some patients exhibited symptoms such as irritant contact dermatitis, allergic contact dermatitis, or an exacerbation of seborrheic dermatitis [14]. A recent systematic review and meta-analysis comparing dutasteride and finasteride suggested that dutasteride offered better efficacy in treating men with AGA compared to finasteride, with no significant difference in adverse reactions [15].

This study has explored incorporating dutasteride into nanostructured lipid carriers (NLCs) with lipids as the main excipients, which may be advantageous for dermal application as the stratum corneum comprises corneocytes surrounded by lipid regions [16]. The lipid matrix of NLCs allows the sustained drug release of many substances [17,18]. Previous studies of topically applied lipid nanoparticles reported lipid film formation on the skin surface, with a subsequent occlusive effect [19,20,21]. This ensures close contact with the stratum corneum, increasing drug penetration into the skin and follicular area. The lipids used in NLCs exhibit a low toxicity, which enhances their tolerability [22].

Chitosan is a chitin derivative, resulting from chitin deamination. The presence of amino groups confers a positive charge [23]. Chitosan is potentially advantageous for skin and hair delivery, as skin and hair are negatively charged and will be attracted to positively charged moieties [24]. In this study, chitosan oligomer (MW: <3 kDa, 85% deacetylation degree) was used. Mittal et al. previously showed that a nanoparticle preparation of antigen ovalbumin with chitosan improved the follicular uptake compared to nanoparticles without chitosan [25]. The surface activity of chitosan (non-conjugated) is low because it has no hydrophobic regions and can be improved by a hydrophobic substituent, using chemical modifications in its glucosidic group [26]. Szymanska and Winnicka reported chemical modification as a strategy for enhancing chitosan stability based on covalent bonds, as well as hydrogen or hydrophobic bonds [27]. A stabilised micellar system prepared using chitosan oligosaccharide (MW 18 kDa) conjugated with stearic acid (CSO-SA) has been studied for brain targeting [28].

In tropical countries, palm and coconut oils which comprise fatty acids such as stearic and lauric acids are widely used as traditional remedies for hair loss [29,30,31,32]. We previously reported [33] that NLCs coated with chitosan oligomer-stearic acid were a promising carrier for dutasteride, reducing hair follicle dermal papilla cell cytotoxicity and providing a sustained-release profile. Lauric acid has been demonstrated to have a potent anti-androgenic activity compared to stearic acid, and had an inhibitory effect on LNCaP cells (Lymph Node Carcinoma of the prostate; a human prostate cancer cell line) [34]. Furthermore, lauric acid produced a greater inhibition of both type I and II 5α-reductase activity than other saturated fatty acids [34]. Liu and Wu [35] prepared lutein-loaded NLC formulations with lauric acid as a solid lipid, but reported that the formulations were unstable even at day 1.

It is hypothesised that coating DST-NLCs with lauric acid (using a chitosan oligomer conjugate with lauric acid), itself having potent anti-androgenic activity, will enhance cell proliferation and hence increase hair growth. This study explores this formulation strategy, aiming to enhance cell proliferation, particularly hair follicle dermal papilla cells, by preparing dutasteride-loaded NLCs coated with lauric acid-chitosan oligomer (CSO-LA). This combination of components represents a novel formulation in which dutasteride, stearic acid (a major component of NLCs) and lauric acid may have additive or synergistic effects in preventing hair loss and promoting hair growth.

## 2. Materials and Methods

### 2.1. Materials

Lauric acid and stearic acid were purchased from Tokyo Chemical Industry (UK). Chitosan oligomer (CSO; MW < 3000 Da) and dutasteride (purity >98.0%) were obtained from Carbosynth (Newbury, UK). Ethanol (96% *v*/*v* analytical grade), acetic acid-d_4_ (99.9 atom %D)_,_ 1-ethyl-3-(3-dimethylaminopropyl), carbodiimide hydrochloride (EDC.HCl), and Sephadex G-50 were obtained from Sigma-Aldrich (Dorset, UK). Acetic acid glacial (analytical reagent), water (HPLC grade), and acetone were purchased from Fisher Scientific (Loughborough, UK). Deuterium oxide (99.9 atom %D) was purchased from Cambridge Isotope Lab. Inc. (Tewksbury, MA, USA). Phosal^®^ 53 MCT and Lutrol^®^ micro 68 were gifted by Lipoid GmbH (Ludwigshafen, Germany) and the BASF Group (Ludwigshafen, Germany), respectively. Deionised water was produced in-house (PURELAB, ELGA, High Wycombe, UK).

### 2.2. Synthesis of Lauric Acid-Chitosan (CSO-LA)

In the presence of EDC.HCl, the free amino groups of chitosan oligomer were synthesised with the carboxyl groups of lauric acid using the method previously described for the synthesis of stearic acid-chitosan, but replacing stearic acid with lauric acid [33]. CSO (1 g) was dissolved in 120 mL of deionised water at 80 °C for 2 h and stirred using a magnetic stirrer. In a separate bottle, 0.5 g of lauric acid (LA) was dissolved in ethanol (80 mL), and EDC.HCl (LA:EDC.HCl molar ratio 1:5 M) was added to LA solutions and heated with stirring at 60 °C for 2 h. The LA solutions were then transferred into CSO solution using a syringe and needle (BD Micro Lance™ 3 Needles 25G, Becton Dickinson, Madrid, Spain) with continuous stirring for 6 h at 80 °C. To reduce the volume, the liquid reaction was evaporated under vacuum (Buchi Rotavapor^®^ R-100, Flawil, Switzerland) at 70 °C. Dialysis was performed for 2 days using a dialysis tube (Molecular weight cut-off 1000 Da, Spectrum Laboratories Inc., New Brunswick, NJ, USA) in order to separate the unreacted EDC.HCl and LA. The product was precipitated using 300 mL of acetone and filtered using a filter paper (Fisherbrand QL100, Fisher Scientific, Loughborough, UK). This process of precipitation/filtration was repeated three times. For further examination, the samples were placed inside a desiccator.

#### 2.2.1. Characterization of CSO-LA Using Fourier Transform Infrared Spectroscopy (FTIR)

Fourier transform infrared (FTIR) spectral measurements were carried out to identify the presence of lauroyl groups in the chitosan oligomer chains, CSO-LA, chitosan oligomer (CSO), and lauric acid (LA). Each sample was analysed with 4 scans from 660 to 4000 cm^−1^ using a PerkinElmer Spectrum 100 FTIR (PerkinElmer, Waltham, MA, USA). The spectrum of CSO-LA was then compared to the spectra for pure CSO and LA.

#### 2.2.2. Characterization of CSO-LA Using ^1^H NMR 

Proton nuclear magnetic resonance spectroscopy (^1^H NMR) analysis was performed for the CSO-LA, CSO, and LA samples in order to confirm the presence of fatty acid chains in the chitosan conjugate. A total of 5 mg of CSO-LA, CSO, and LA was dissolved individually in 1 mL of deuterium oxide (D_2_O) with the addition of one drop of acetic acid-d_4_ (for the chitosan-based sample only). The ^1^H NMR spectra were obtained using an NMR Bruker Avance 500 spectrometer (Bruker Instruments, Billerica, MA, USA).

#### 2.2.3. Determination of Amine Group Substitution Using the Ninhydrin Assay

The percentage of free amino groups in the CSO-LA conjugate was calculated using the ninhydrin calorimetric reaction, as previously described [33], assuming that the d-glucosamine slope has 100 percent amine groups, and then CSO and CSO-LA were identified by comparing each slope. The absorbance of the reaction solutions (purple colour) was determined at 570 nm using an Agilent Cary 100 UV-Vis Spectrometer (Agilent Technologies UK Ltd., Wokingham, UK).

### 2.3. Preparation of Nanostructured Lipid Carriers (NLCs)

Dutasteride-loaded nanostructured lipid carriers (DST-NLCs) were prepared using the ultrasonic melt-dispersion technique as described previously [33]. Dutasteride (17.5 mg), stearic acid (300 mg), and Phosal^®^ 53 MCT (75 mg) were weighed in a glass vial. Lutrol^®^ micro 68 (150 mg) was added to another glass vial with deionised water (10 mL, ~pH 5.6). Both glass vials were heated separately at 80–90 °C in a water bath, mixed, and homogenised using an IKA Ultra Turrax T25 (IKA Werke, Staufen, Germany) at 19,000 rpm for 10 min. The hot dispersion was probe sonicated (MSE Soniprep 150, MSE, East Sussex, UK) at 18 W for 5 min. A total of 2 mL of hot dispersion was quickly added using a syringe (needle: 25 gauge, 5/8th inch) into 10 mL of cold water (4–8 °C) and stirred using a magnetic stirrer for 10 min to obtain nanoparticles. Blank NLCs were prepared following the same procedures (with the exclusion of dutasteride). All the nanoparticle formulations were stored at 4–8 °C for 24 h before being coated and/or characterised.

Since dutasteride is non-fluorescing, Coumarin-6 (Cou-6) was employed as a fluorescent model drug and incorporated into the NLCs in order to observe the cell and skin uptake of the carrier. The molecular weights for dutasteride and Coumarin-6 are 528.53 and 350.4 g/mol, respectively. Both are insoluble in water. Due to the comparable hydrophobicity characteristics of the two compounds (Log P dutasteride = 5.09 and Log P Cou-6 = 6.06), Coumarin-6 was considered suitable for use in this study. A total of 0.2 mg Cou-6 with the same amounts of stearic acid, Phosal^®^ 53 MCT, and Lutrol^®^ micro 68 outlined above were used, and Cou-6-loaded NLCs (Cou-6-NLCs) were prepared as described for the DST-NLCs. 

#### Preparation of NLCs coated with lauric acid-chitosan oligomer (CSO-LA)

A total of 250 µL of CSO-LA (5.0 mg/mL in 0.2% acetic acid solution) was added to 5 mL of NLC preparations using a syringe (needle: 25 gauge, 5/8th inch), with constant stirring for 10 min. The prepared CSO-LA was declared as 5% CSO-LA. Cou-6-NLCs were coated with 5% CSO-LA using the same procedure.

### 2.4. Characterization of NLCs, Uncoated and Coated with CSO-LA

#### 2.4.1. Measurement of Particle Size Distribution and Zeta Potential and Determination of Morphology

The size distribution and zeta potential of NLCs were determined as the hydrodynamic diameter, polydispersity index (PDI), and zeta potential (ξ) using a Zetasizer Nano ZS (Malvern Instruments Ltd., Malvern, UK) at 25 °C with zeta potential capillary cells. Measurements were performed 3 times without dilution, and mean values were taken. The physical stability of DST-NLCs was monitored, examining changes in the particle size distribution and zeta potential with time. Each formulation was kept in a refrigerator at 4–8 °C. The particle size distribution (mean size and PDI) and zeta potential were compared at day 1 and day 180.

The morphology of the nanoparticles was determined using transmission electron microscopy (TEM). One drop of each sample was placed on a copper grid. After 2 min, one drop of 1% uranyl acetate was placed onto the grid for negative staining. The grid was dried at room temperature and observed using TEM (Philips/FEI CM120 Bio Twin, FEI Company, Eindhoven, The Netherlands).

#### 2.4.2. Entrapment Efficiency and Drug Loading 

The entrapment efficiency and drug loading were calculated following the separation of free and entrapped drug using Sephadex^®^ gel G-50 as a mini column and centrifugation at 1000 rpm, as described previously [33]. However, it was not possible to achieve effective the separation of coated preparations using the Sephadex-G50 column, despite repeated experiments, and consequently the entrapment efficiency could be calculated only for the uncoated DST-NLCs. High-performance liquid chromatography (HPLC) with U*V*/*V*is detector (Agilent 1100 Series, Agilent Technologies Inc., Santa Clara, CA, USA) at a 241 nm wavelength was used to quantify the total dutasteride in NLCs and the encapsulated drug following centrifugation, as previously described [33]. The analytical column, Synergi™ 4 µm Polar-RP 80 Å (250 × 4.6 mm) as the stationary phase and a mobile phase of acetonitrile and 0.1% trifluroacetic acid (TFA) in HPLC-grade water (70:30 by volume) were used. The injection volume was 30 µL, with a 1 mL/min flow rate.

The entrapment efficiency and drug loading for Cou-6-NLCs was also calculated. Cou-6 was quantified using HPLC as described for dutasteride, except that the wavelength for the detection of Cou-6 was 466 nm.

### 2.5. In Vitro Drug Release and Skin Permeation

**Figure 1:**
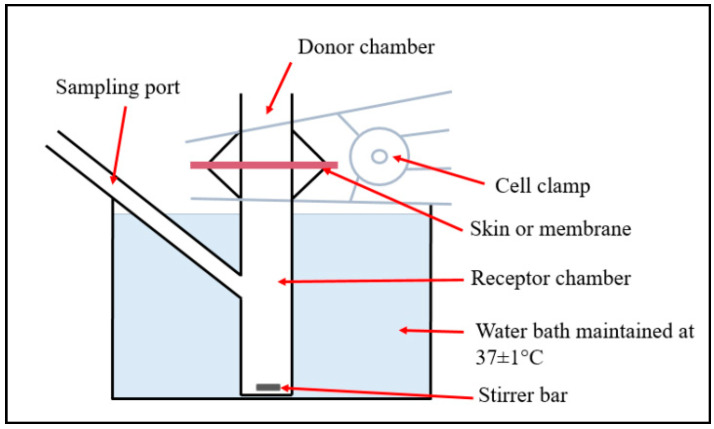
Diagram of the Franz diffusion cell used for the in vitro drug release and permeation studies.

The in vitro drug release and skin permeation were determined using a Franz diffusion cell (PermeGear, Hellertown, PA, USA), as previously described [33]. The final total drug concentration in the DST-NLCs was 0.29 mg/mL. The total dutasteride content of 250 uL of DST-NLCs or DST-NLCs coated with CSO-LA in 5 mL of 2% SDS in PBS at pH 7.4 was measured using HPLC and found to be approximately 67 and 61 µg, respectively. For the release study, a 0.45 µm HA nitrocellulose membrane (MF™ Membrane Filters, Merck Millipore, Cork, Ireland) and 0.29 mg/mL of dutasteride in 70% ETOH were applied. For the permeation study, pig ear skin (freshly slaughtered pigs for food consumption from Farnborough, UK) was used as shown in Figure 1. The ears were washed with deionised water, and the hair was carefully cut with scissors. The subcutaneous tissue was removed. The average thickness of the skin was ~0.5 mm, which was cut and frozen (−20 °C) for later use. The Franz diffusion cell (surface area = 0.64 cm^2^) was maintained at 37 °C and stirred using a magnetic stirrer (600 rpm). Due to the very low solubility of dutasteride in water (0.038 ng/mL), for the release study 2% sodium dodecyl sulfate (SDS; required to ensure sink conditions as determined in preliminary experiments, data not shown) in phosphate-buffered saline (PBS 7.4) with the addition of 0.02% sodium azide for the permeation study was used. DST-NLCs (250 µL), uncoated and coated with 5% CSO-LA, were pipetted in the donor chamber. Samples (200 µL) were taken at 0, 0.25, 0.5, 1, 2, 3, 6, 8, 12, 24, 30, 36, and 48 h from the receptor chamber, with the medium replaced with 200 µL of fresh buffer. The samples were injected into the HPLC to quantify the drug released.

The skin was placed between the donor and receptor chambers to assess the in vitro permeation and was allowed to rest for 1 h. Then, 200 μL samples were taken from the receptor chamber at 0, 0.25, 0.5, 1, 2, 3, 6, 8, 12, 24, 30, 36, and 48 h, and replaced with 200 μL of fresh buffer. The formulation in the donor compartment was carefully collected using a pipette tip and a tissue paper. A total of 2 mL of ethanol was used to collect the remaining formulation in the donor compartment and to rinse the upper part of the skin. The stratum corneum was removed after 48 h using adhesive tape (25 mm × 20 mm; 3 M Scotch magic™ Tape, Saint Paul, MN, USA) 10 times. The tapes were collected and 1 mL of ethanol was applied, following bath sonication for 1 h. Using a scalpel, the epidermis/dermis was cut into small pieces and added to 0.5 mL ethanol for 24 h and sonicated for 1 h. A 0.22 μM polyethersulfone (PES) membrane syringe filter (Millex^®^ GP, Merck Millipore, Cork, Ireland) was used to filter the solutions before injection into the HPLC for drug quantification.

### 2.6. In Vitro Cytotoxicity and Cell Proliferation Studies on Hair Follicle Dermal Papilla Cells

In vitro cytotoxicity and cell proliferation studies on normal hair follicle dermal papilla cells were undertaken as described previously [33]. Cultured human hair follicle dermal papilla cells (HFDPCs) (Promo-Cell, Heidelberg, Germany) from passage 4 were seeded (7.5 × 10^4^ or 3.75 × 10^4^ per mL) onto a 96-well plate (Nunc, Wiesbaden, Germany) and grown to a confluence of 60–70% for 24 h. The MTT assay for cytotoxicity and proliferation was conducted for 5 days. Treatment was conducted with dutasteride alone (dutasteride was dissolved in DMSO at 100 mM and serial dilutions were prepared from 100 to 1.6 µM), dutasteride in NLCs uncoated or coated with 5% CSO-LA (dutasteride concentration 12.5–100 µM), and empty NLCs uncoated and coated with CSO-LA, with minoxidil (100 µM) as a positive control. Absorbance was determined spectrophotometrically at 570 nm using a microplate reader (SpectraMax^®^M2e, Mol. Devices, San Jose, CA, USA). The results are expressed as percentages of untreated controls in four cultures. The reported values are the means ± SD.

### 2.7. In Vitro Skin Irritation Study Using EpiDerm™ SIT 3D Reconstructed Human Epidermis (RhE)

The skin irritation test using 3D EpiDerm™ was based on the UN Globally Harmonised System of Classification and Labelling (GHS) Category 2 and followed OECD Test No. 439. The procedure was performed based on the protocol supplied by the test manufacturer (MatTek In Vitro Life Science Laboratories, Bratislava, Slovakia). The percent cell viability was measured by comparing the formulations with the negative control (Dulbecco’s Phosphate Buffer Saline; DPBS). Tissues were dosed with 30 μL of test substances (DST-NLCs, NLCs, CSO-LA-coated DST-NLCs, CSO-LA-coated NLCs), a positive control (5% SDS), and a negative control (DPBS) on the day of treatment. For 60 min, the tissues were exposed to the formulations and then rinsed with DPBS 15 times, transferred to a fresh assay medium, and incubated for 42 ± 2 h at 37 ± 1 ° C, 5 ± 1% CO_2_. The tissues were blotted and the MTT assay was carried out. Formazan products were collected from the cells after 24 h using isopropanol, and read at 570 nm using the microplate reader (SpectraMax ^®^ M2e, Molecular Devices, San Jose, CA, USA).

### 2.8. Skin and Cell Uptake of Cou-6 NLCs Uncoated and Coated with CSO-LA

#### 2.8.1. Skin Uptake of Cou-6 NLCs

In this experiment, Cou-6 was used in the NLCs, with a comparable amount of Cou-6 (0.003 mg/mL) diluted as a control with 70% ethanol. The Franz diffusion cell set-up was identical to the research on permeation, as outlined above. The pig ear skin was placed into Franz diffusion tubes, and uncoated or coated with CSO-LA Cou-6 NLC formulation was pipetted into the donor chamber. The skin was removed every 6, 12, and 24 h. The removed skin was further frozen at −30 °C in a Leica CM1850 cryostat (Leica Microsystems, Wetzlar, Germany) before being fitted to the cryostat chuck using the compound OCT (optimum temperature cutting) (VWR International Ltd., Belfast, UK). The skin was cryotomed at a thickness of 50 μm at −30 °C and the skin slices were transferred directly to microscope adhesion slides (Adhesion slides, SuperFrost Plus, VWR, Belfast, UK) and visualised using an inverted fluorescence microscope (EVOS ^™^ FL Imaging System, Life Technologies, Carlsbad, CA, USA) with a EVOS ™ DAPI (4′,6-diamidino-2-phenylindole) light cube filter without extracting the stratum corneum.

#### 2.8.2. Cell Uptake of Cou-6 NLCs

For the analysis of cell uptake, the same amount of NLCs was used on the hair follicle dermal papilla cells to ensure that the concentration of Cou-6-loaded NLCs did not affect the cell viability. A total of 1 mL of HFDPCs with a density of 3.75 × 10^4^ cells/mL was transferred to a 12-well plate and left for 24 h. Cou-6-NLCs uncoated and coated with CSO-LA and CSO-LA and Cou-6 in ethanol were applied to a 12-well plate on the day of testing. The cell media were drained at 1, 3, 6, and 12 h, and washed with PBS at pH 7.4 before imaging using a fluorescence microscope, with a 10× magnification EVOS ™ DAPI light cube filter.

### 2.9. Statistical Analysis

A statistical analysis (IBM SPSS Statistic 23) of all data was performed using a t-test or one-way ANOVA and Tukey’s post-hoc test. A *p*-value smaller than 0.05 was considered statistically significant.

## 3. Results and Discussion

### 3.1. Synthesis and Characterization of CSO-LA

#### 3.1.1. ^1^H NMR, FTIR, and Degree of Substitution

**Figure 2:**
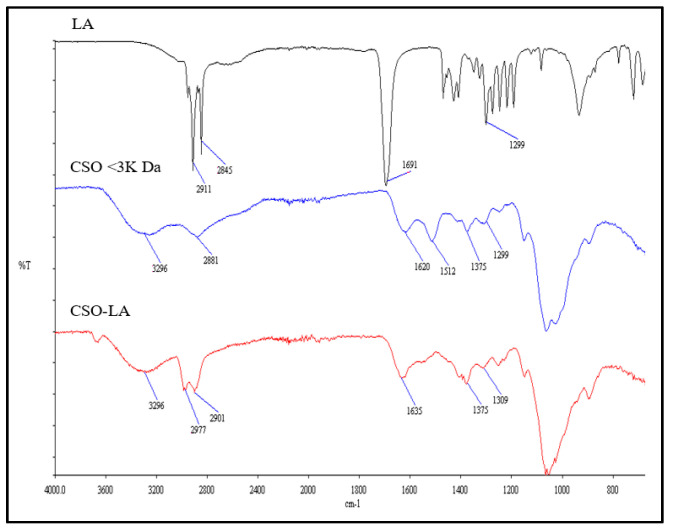
FTIR spectra of lauric acid (LA), chitosan oligomer (CSO), and chitosan oligomer-lauric acid (CSO-LA).

The CSO-LA formation was confirmed using both FTIR and ^1^H NMR. There were some differences in the IR spectrum of the chitosan oligomer-lauric acid resulting from the interaction between the carboxylic group of lauric acid and the amine group of the chitosan oligomer compared with the spectra of lauric acid alone and the chitosan oligomer (Figure 2).

The IR spectrum of CSO (without conjugation) had an absorption peak at 1620 cm^−1^ due to the secondary amine (amide I band) stretching of C=O. The IR spectrum of CSO-LA revealed two moved peaks corresponding to the *N*-lauroyl (amide I and II bands, respectively) at around 1635 and 1529 cm^−1^. The increase in C-H absorption at about 2977 and 2901 cm^−1^ suggested that the lauroyl chain was present. The broad peak at around 3296 cm^−1^ indicates the chitosan oligomer group -OH. The other peak showing the presence of the lauric acid carboxyl groups (-COOH) is at ~1691 cm^−1^. After lauric acid was conjugated to a chitosan oligomer, the peak at about 1691 cm^−1^was reduced, indicating that the lauric acid had reacted with the chitosan oligomer. 

**Figure 3:**
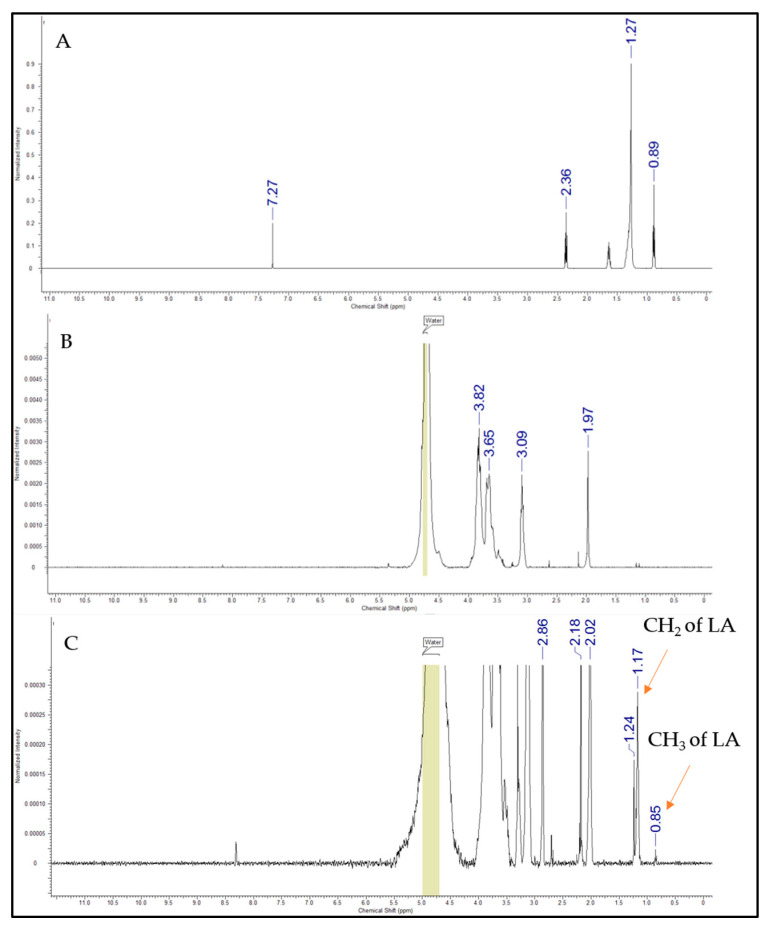
^1^H NMR spectra of lauric acid (**A**), chitosan oligomer (**B**), and CSO-LA (**C**).

Chemical shifts occurred within the ^1^H NMR spectra (Figure 3), which represented the laurate group’s methyl and methylene hydrogen. The chemical changes shown at 0.85 ppm indicated the protons of lauric acid CH_3_, while the peaks at 1.17–1.24 ppm corresponded to the protons of the CH_2_ group of the acyl chain. An additional peak at 8.4 ppm appeared for CSO-LA, suggesting that amide bonds were formed, as supported by Zhao et al. [36]. The remaining peaks were due to hydrogen from the chitosan oligomer. These results indicated the successful conjugation of lauric acid with the chitosan oligomer and were in accordance with the findings of Zhao et al. [36], who successfully combined LA with CSO for titanium surface functionalization to enhance the osteoblast functions and inhibit bacterial adhesion.

#### 3.1.2. Ninhydrin Assay of d-Glucosamine, CSO and CSO-LA

**Figure 4:**
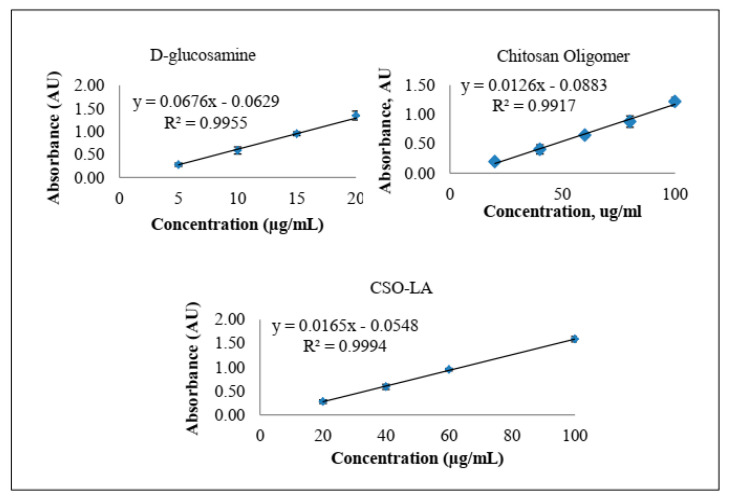
Absorbance versus concentration plots from the ninhydrin assay of d-glucosamine, CSO, and CSO-LA (*n* = 3, mean ± SD).

Using the ninhydrin assay (Figure 4), the slopes were 0.0126 and 0.0092 for chitosan oligomer (CSO) and CSO-LA, respectively. By comparing the slopes from glucosamine, CSO, and CSO-LA, the degree of substitution (%DS) of CSO-LA was calculated as 6.2%.

The value for %DS of CSO-LA was similar to that previously reported (6.5%) for the conjugation of CSO with stearic acid [33] at the same molar ratio of fatty acid: EDC.HCl.

### 3.2. Preparation and Characterization of DST-NLCs, Uncoated and Coated with CSO-LA

#### 3.2.1. Particle Size Distribution and Surface Charge of Nanoparticles

DST-NLCs were coated with CSO-LA, as shown in Table 1 (day 1). The mean sizes of the uncoated NLCs and those coated with CSO-LA were not significantly different (*p* > 0.05). There was no substantial difference in PDI (*p* > 0.05) between the uncoated NLCs and those coated with CSO-LA, with all PDI values below 0.3 suggesting a relatively narrow size distribution.

The zeta potential of DST-NLCs changed from negative to positive on coating (*p* < 0.05), with CSO-LA adsorbed on negatively charged DST-NLCs, producing net positively charged nanoparticles (Table 1), with the value increasing as a function of the CSO-LA concentration. These results suggest a strong electrostatic interaction between the negative surface of DST-NLCs and the positively charged CSO-LA. The zeta potential determined at day 1 and day 180 (*p* > 0.05) did not indicate any significant difference.

The uncoated and 5% CSO-LA-coated DST-NLCs had significantly different mean particle sizes at day 1 and at day 180 (*p* < 0.05). For both formulations, there was no significant difference (*p* > 0.05) between day 1 and day 180 in terms of the PDI value (a measure of the size distribution of the nanoparticles). The particle size distribution (mean size and PDI) was also measured at day 30 and day 60 for both preparations stored at the same conditions; the measured values were all intermediate between those measured at day 1 and at day 180 (data not shown)

Other studies have also shown the electrostatic interaction between positively charged chitosan and negatively charged nanoparticles (in solid lipid nanoparticles or in nanostructured lipid carriers) [35,36,37,38,39]. DST-NLCs coated with CSO-LA would not pass down a Sephadex gel column despite repeated attempts, preventing the calculation of drug entrapment and drug loading. Therefore, the entrapment efficiency was only measured for uncoated DST-NLCs and was found to be 97.3 ± 1.2% on day 1, with no significant difference compared to day 180 (*p* > 0.05). The drug loading of DST-NLCs was found to be 3.49 ± 0.10% at day 1.

Table 2 shows the effect of coating Cou-6-NLCs with 5% CSO-LA on the size distribution and surface charge. The mean size did not increase (*p* > 0.05), whilst the zeta potential changed from negative to positive on coating. The PDI for the Cou-6-NLCs with and without 5% CSO-LA was below 0.3, indicating a narrow size distribution. The values for the mean particle size and zeta potential for the uncoated or coated Cou-6-NLCs with 5% CSO-LA were not significantly different (*p* > 0.05) from those of the uncoated DST-NLCs and 5% CSO-LA (Table 2). Such results suggest that Cou-6 was ideal as a model for dutasteride for subsequent NLC formulation for uptake studies.

#### 3.2.2. Morphology of Dutasteride-Loaded NLCs Uncoated and Coated with 5% CSO-LA

**Figure 5:**
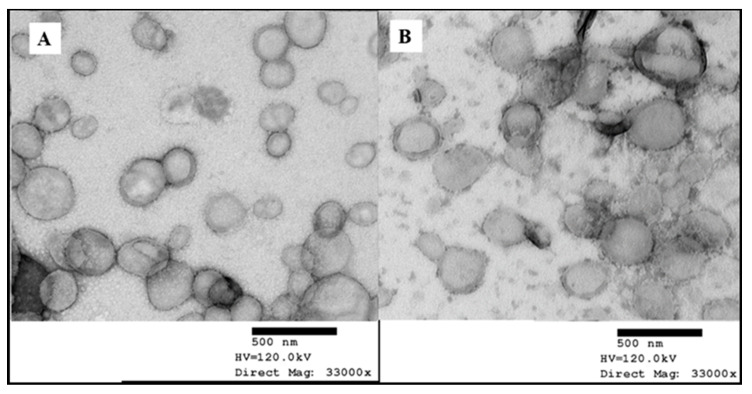
Transmission electron micrographs of DST-NLCs, uncoated (**A**) and coated with 5% CSO-LA (**B**) (stained with 1% uranyl acetate).

In the present research, the morphology of nanoparticles was explored using TEM images. The uncoated DST-NLCs and the CSO-LA-coated nanoparticles were visualised as approximately 200 to 250 nm mono-dispersed, nearly spherical nanoparticles (Figure 5).

### 3.3. In Vitro Dutasteride Release from DST-NLCs Uncoated and Coated with CSO-LA

**Figure 6:**
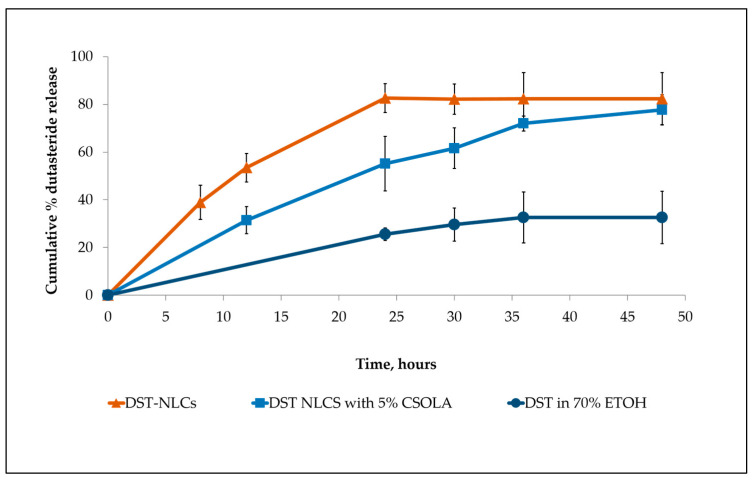
Drug release from the uncoated DST-NLC, coated with 5% CSO-LA and dutasteride in 70% ethanol (*n* = 3, mean ± SD).

From the HPLC analysis, the linear regression of the dutasteride data showed a good linearity ranging from 3 to 100 μg/mL. The limit of detection (LOD) and limit of quantification (LOQ) were 0.38 and 3 μg/mL, respectively. The release profiles of both nanoparticle formulations are shown in Figure 6, with a faster drug release exhibited by uncoated DST-NLCs (*p* < 0.05). DST-NLCs without coating showed an approximately 80% drug release at 24 h, while nanoparticles coated with CSO-LA released 55% over the same time period. The presence of the polymer in the outer regions of the DST-NLCs coated with CSO-LA significantly reduced the amount of drug released (*p <* 0.05) at 12, 24, and 30 h. Dutasteride in ethanolic solution shows the slowest drug release. This could be due to the limited dutasteride solubility in the receptor medium [37].

### 3.4. In Vitro Skin Permeation

No dutasteride could be detected in the receptor chamber for both formulations analysed by HPLC. The amounts of dutasteride for DST-NLCs and 5% CSO-LA in the skin (Table 3) were significantly different (*p* < 0.05). This is similar to the results reported for DST-NLCs coated with CSO-SA [38], where no dutasteride had permeated through the skin after 48 h. This may be due to the difference in surface charge between the chitosan and skin epithelium [39], and the bioadhesive property of chitosan [40]. This agrees with the findings of Siqueira et al. [38], whereby the amount of benzophenone-3 permeating to the lower receptor chamber was reduced for chitosan-coated polymeric nanocapsules compared to a simple formulation containing pure drug.

### 3.5. In Vitro Cytotoxicity on Hair Follicle Dermal Papilla Cells

**Figure 7:**
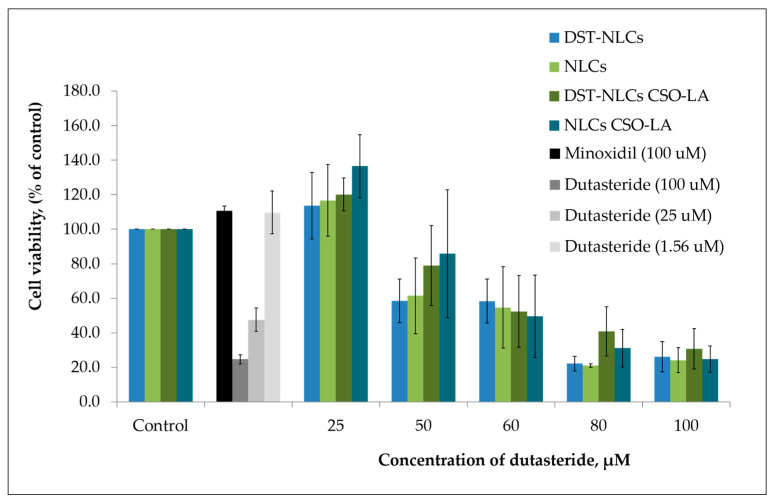
Cytotoxicity of dutasteride alone, empty NLCs/NLCs-CSO-LA, and DST-NLCs coated and uncoated with 5% CSO-LA on the hair follicle dermal papilla cells after 5 days (*n* = 4, mean ± SD). The amount of dutasteride in the NLCs corresponds with that of the pure drug.

There was a significant difference (*p* < 0.05) in the cytotoxicity values (half maximal inhibitory concentration; IC_50_) among the tested compounds (Figure 7). The IC_50_ was 10.4 ± 1.82 μM for dutasteride. The IC_50_ values of the DST-NLCs for both uncoated and coated with CSO-LA were much higher, up to 58.1 ± 13.0 μM and 58.9 ± 14. 8 μM. NLCs alone (without dutasteride) uncoated and coated with CSO-LA showed IC_50_ values of 58.1 ± 13.0 μM and 56.2 ± 12.6 μM, respectively. There were significant differences in the IC_50_ values for dutasteride (alone) with DST-NLCs, and NLCs coated with CSO-LA (*p* < 0.05).

The maximum non-toxic concentrations (MNTC, EC_90_—concentration of a formulation or drug inhibiting 10% of the cell) were 38.1 ± 13.1 µM and 39.3 ± 11.5 µM for the uncoated DST-NLCs and those coated with CSO-LA, respectively (*p >* 0.05). The MNTCs for NLCs uncoated and coated with CSO-LA were also not different (*p >* 0.05), being respectively 42.3 ± 12.4 µM and 39.3 ± 11.5 µM. 

However, the MNTC (EC_90_) for dutasteride alone without nanoparticles was much lower, at 2.1 ± 0.2 µM. This finding suggests that the dutasteride in nanoparticles coated with CSO-LA could be applied at a higher concentration compared to dutasteride without nanoparticles. The dutasteride delivery in nanoparticles increased the MNTC (EC90) over 20-fold at a higher concentration than dutasteride without nanoparticles, suggesting that the slow release of the drug from NLCs might reduce the toxicity to cells. The result was supported by previous research [37], which found that the cell viability of 5α-reductase inhibitors entrapped in surface-modified liquid crystalline nanoparticles on HaCat cells was reduced when the concentration increased from 10 to 500 µg/mL.

Minoxidil at 100 µM, as a positive control, produced cell proliferation by 110.6 ± 2.8%. Some of the nanoparticulate formulations with 25 μM of dutasteride produced cell proliferation; the greatest proliferation was shown by DST-NLC and NLCs (empty) coated with CSO-LA, each with 120.2 ± 9.5% and 136.6 ± 18.3%. It is interesting to note that the NLCs (without dutasteride) also caused the proliferation of the hair follicle dermal papilla cells when compared to the control for those coated with CSO-LA. These findings indicate that stearic acid, a major component of NLCs in combination with CSO-LA used as a coating material, promotes cell growth. Previous research [34,41] has reported that stearic acid has a less potent and limited activity on 5α-reductase inhibition. However, by incorporation into a formulation with lauric acid (CSO-LA), it has induced a higher proliferation of the hair follicle dermal papilla cells.

### 3.6. In Vitro Skin Irritation

**Figure 8:**
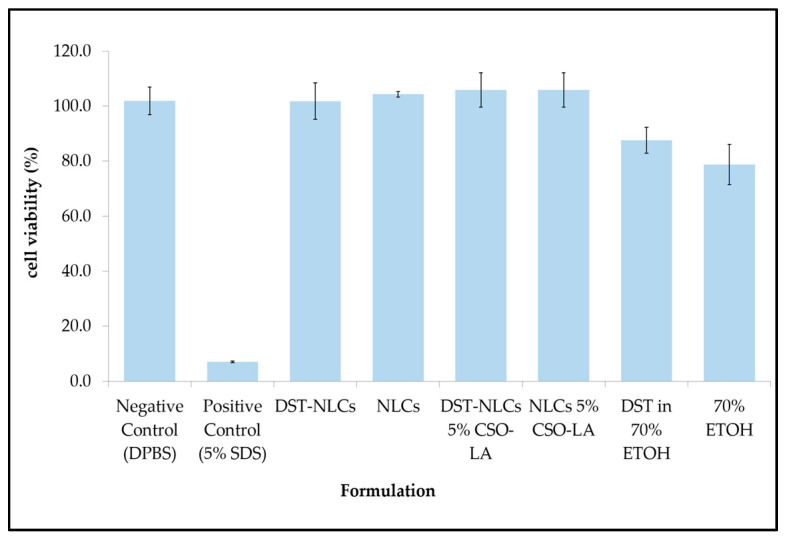
Skin irritation (cell viability) of different formulations on 3D reconstructed human epidermis (RHE) (*n* = 3, mean ± SD).

In order to explore the possibility of irritancy resulting from the formulations and their constituents, 3D reconstructed human epidermis (EpiDerm™ RHE) was employed. Based on the validated protocol provided by the manufacturer, a cell viability less than 50% indicates the irritant potential of ingredients classified as Globally Harmonised System (GHS) category 2 or Irritant (I). All the formulations (nanoparticle or components alone) showed no irritation, with a mean cell viability greater than 50% (Figure 8).

There was a significant difference (*p* < 0.05) between the positive control (5% sodium dodecyl sulfate, producing 7% cell viability), which showed irritancy, and all formulations including the negative control. The IC_50_s for the compositions of nanoparticles that were coated or uncoated were not substantially different from those of the negative control (DPBS) (*p* > 0.05).

The IC_50_ values for 70% ethanol and 0.29% dutasteride in 70% ethanol (78.7 ± 7.3% and 87.6 ± 4.7%, respectively) were significantly different (*p <* 0.05) compared to the negative control (DPBS). This result has shown that having ethanol (even at 70%) as a carrier in any topical products resulted in a reduced cell viability, in agreement with previous research using rabbit Draize test data [42]. These findings also agree with Lee et al. [43], who reported that a formulation containing 70% ethanol tested on EpiDerm™ caused approximately 40% of cells to die. Moreover, adverse effects such as dryness, erythema, and desquamation have been reported for the repeated topical application of ethanol/propylene glycol/water-based minoxidil solutions [44].

Comparing the cell viability of DST-NLCs and DST in 70% ethanol, delivering dutasteride in the nanoparticles promoted a higher cell viability (*p* < 0.05) compared to when ethanol was employed as the vehicle. All the formulations, coated or uncoated, with or without DST, showed no irritation (approximately > 96% cell viability)—i.e., dutasteride loaded into NLCs, either uncoated or coated with CSO-LA, produced no irritancy on the skin. Currently, dutasteride is marketed in soft gelatin capsules for oral administration. It has been claimed that dutasteride soft capsules can cause irritation (Skin Irritation Category 2) if the capsule is broken [45]. Having dutasteride in the nanoparticle system developed in this study, with Generally Recognised as Safe (GRAS) ingredients, could reduce the irritancy as dutasteride is released slowly on the skin, as demonstrated by the release and skin irritation results.

### 3.7. Skin Uptake of Cou-6 Delivered in Ethanol and Cou-6-Loaded NLCs, Uncoated and Coated with 5% CSO-LA 

**Figure 9:**
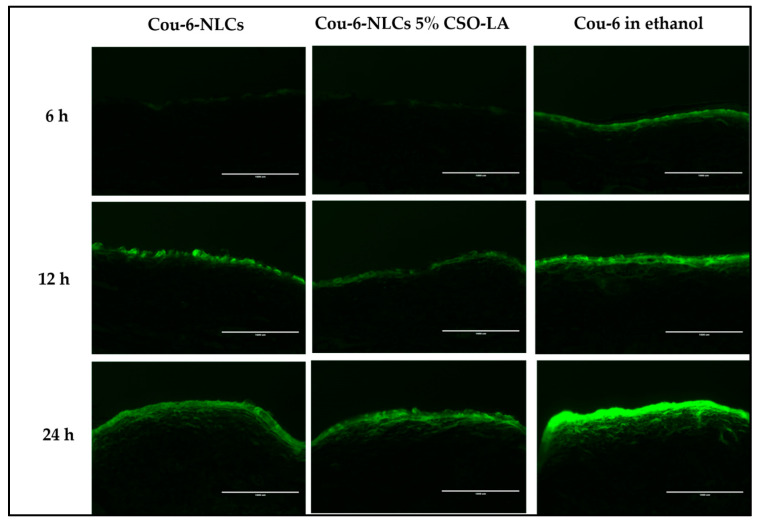
Skin uptake of Cou-6 in full-thickness pig ear skin (cryostat set at 50 µm thickness) from Cou-6-loaded NLCs, uncoated and coated with CSO-LA, and Cou-6 in ethanolic solution over 24 h (scale bar = 1000 µM).

Figure 9 shows the skin uptake of Cou-6-NLCs, uncoated and coated with 5% CSO-LA, and Cou-6 in ethanol at different time points using a qualitative assay. Due to the limited amount of Cou-6 that can be included in the formulations, leading to levels below the LOQ of the test, the dye could not be quantified in the skin. 

The Cou-6 was taken up by the skin in every formulation in the upper skin layer in a time-dependent manner, with a higher intake at 12 and 24 h than at 6 h, especially in the upper skin region. The coloration was also seen in the deeper skin layer. At 6 h, a lower fluorescent dye intensity was seen in the skin from the Cou-6-NLCs uncoated and coated with CSO-LA, which suggested slow release from the NLCs at first, although the intensity increased the longer the dye exposure was provided to the skin. Cou-6 in ethanolic solutions displayed the highest fluorescent strength compared with nanoparticle formulations at 6 h, as a result of direct interaction between the skin and the dye.

The NLCs had a mean diameter of approximately 190 nm. Lin et al. prepared diphencyprone-charged NLCs with a hydrodynamic diameter of approximately 208 to 265 nm; this size range provided the localisation of NLCs in follicles and intercellular lipids of the stratum corneum after 6 h of percutaneous in vivo absorption [46].

Further research examining the deposition of nanoparticles in mammalian skin found that nanoparticles with a mean size of 20 to 200 nm did not penetrate beyond the superficial layers of the barrier, even at the smallest size (20 nm), with no nanoparticles penetrating into the deeper layer of the skin [47]. This showed that nanoparticles could not penetrate the barrier to the skin, but could be useful as reservoirs on the skin surface to control the release of drugs over time [47]. Patlolla et al. also stated that nanoparticles do not cross the skin but may permeate into the stratum corneum and release the drug into the upper epidermis in a controlled manner [48].

Nanoparticles, however, tend to move and persist in the hair follicles [49]. An earlier study showed that nanoparticles with a size of 320 nm had a high accumulation of drugs after massage in the transfollicular region, and were retained in the hair follicle for up to 10 days. [50]. Massaging was not implemented in this study, and the intensity of Cou-6 can mostly be seen in the upper skin layer. If massaging is applied, the nanoparticles may go into the transfollicular region and stay in the hair follicles for longer. However, in the skin samples examined in this study, no transfollicular region could be identified.

### 3.8. Cell Uptake of Cou-6 Delivered in Ethanol and Cou-6-Loaded NLCs, Uncoated and Coated with 5% CSO-LA

**Figure 10:**
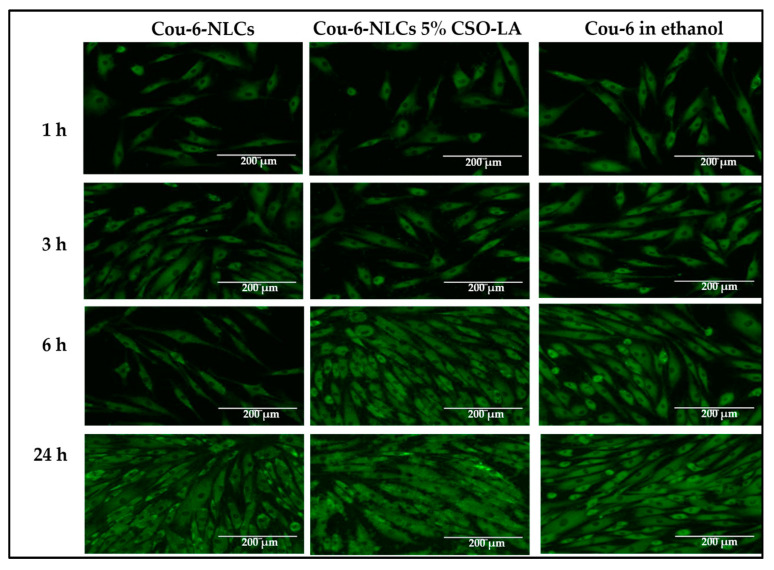
Cell uptake of Cou-6 in hair follicle dermal papilla cells from Cou-6-loaded NLCs, uncoated and coated with 5% CSO-LA, and Cou-6 in ethanolic solution (scale bar = 200 µM).

Figure 10 indicates the absorption of Cou-6 in ethanolic solution and NLCs in the dermal papilla cells of hair follicles at different time points. The dye could not be quantified using HPLC or a UV spectrophotometer (lower than LOQ) due to the small amount of Cou-6 taken up by the cells. There was a similar pattern in the cell uptake as for the skin uptake (Section 3.7), with Cou-6 being taken up in a time-dependent manner in all formulations. There was no large difference in the fluorescence dye intensity within cells from the solution or nanoparticles. At the latest time point, the level was highest for all formulations, suggesting that the cells took up Cou-6. Considering the near proximity of the Log P values of Cou-6 and dutasteride, Cou-6 can be considered a good model for studying the cell absorption of these systems.

The findings here are supported by Rivolta et al. [51], who prepared Cou-6-loaded solid lipid nanoparticles with a hydrodynamic diameter of 116 nm and a PDI of 0.31 and demonstrated their uptake by alveolar epithelial cells. There was no co-localisation with the dye in the cells. Similar findings have been reported for Cou-6-loaded SLNs, with increasing amounts of Cou-6 being taken up by A549 lung cancer cells with increased concentration and time [52]. From these results, uncoated and coated Cou-6-loaded NLCs provide an effective model for studies of dutasteride-loaded NLCs with similar physical characteristics (size distribution and zeta potential).

### 3.9. Comparison of Findings with an Earlier Study (Noor et al., 2017) using DST-NLCs Coated with Chitosan-Stearic Acid 

Table 4 compares the properties of DST-NLCs and NLC coated with 5% CSO-LA and 5% CSO-SA. The data for NLCs coated with CSO-SA was previously reported by us [33] in a paper which used a Design of Experiments approach to optimise DST-NLCs in order to achieve a small mean particle size, narrow PDI, and high drug entrapment efficiency. In the current paper, a DST-NLC system coated with 5% CSO-LA was prepared and characterised, then further studied in terms of the in vitro performance with a focus on cytotoxicity, skin irritation, and cell and skin uptake.

Noor et al. [33] compared optimised DST-NLCs coated with CSO conjugated with stearic acid (CSO-SA) or CSO (chitosan oligomer without conjugation). DST-NLCs were coated with different percentages of coating material: 5% CSO-SA, 10% CSO, and 5% CSO (without conjugation). The data showed that 5% CSO-SA was the optimum concentration of those tested. DST-NLCs coated with CSO (no conjugation) showed aggregation over time at 4–8 °C for 30 days, whilst DST NLCs coated with 5% CSO-SA were stable for up to 60 days. 

The mean particle size of DST-NLCs coated with CSO-LA was significantly smaller (*p* < 0.05) compared to the particles coated with CSO-SA, but did not differ significantly from the uncoated NLCs (*p* > 0.05). This suggests that the shorter chain of lauric acid compared to the stearic acid in the coating results in a thinner coating and/or a better reproducibility of production or dispersibility, as the variability in the mean size is reduced with the CSO-LA coating compared to CSO-LA. The zeta potential and PDI for NLCs prepared with both coatings were not significantly different (*p* > 0.05). The drug release profiles for the two coated nanoparticle preparations were similar; both showed a similar (*p* > 0.05) dutasteride release at 24 h, which was significantly slower than for the nanoparticles without a coating (*p* < 0.05). Franz diffusion cells were used to study the dutasteride permeation from all formulations. Between both coatings, there was no significant difference for permeation (*p* > 0.05). The maximum non-toxic concentration, (EC_90_), was not significantly different for all formulations (*p* > 0.05), and all were significantly higher (*p* < 0.05) than that of DST alone, indicating that drug incorporation into the nanoparticle formulations would allow higher drug concentrations to be applied in vivo.

The physical characteristics and cytotoxicity of the NLCs coated with CSO-SA and CSO-LA were thus very similar, with one coating material not offering particular advantages over the other. However, for the current study, the coating material was selected to be CSO-LA and the in vitro performance was studied further ahead of in vivo studies. It is anticipated that this formulation could potentially improve in vivo performance, compared to CSO-SA, as lauric acid itself has anti-androgenic activity [34] that could potentially enhance the performance of the product by increasing the cell proliferation in vivo.

The results of the cytotoxicity study (Figure 7) showed significantly reduced toxicities for dutasteride in DST-NLCs, uncoated or coated with CSO-LA, with evidence of proliferation at some concentrations. Previous research [36] has also demonstrated that the cell adhesion, cell viability, intracellular alkaline phosphatase activity, and osteoblast mineralization capability were significantly improved when titanium substrates were cultivated on the surface of chitosan–lauric acid, indicating that the inclusion of this material within our formulation may bring additional benefits in vivo.

## 4. Conclusions

The main objective of this study was achieved. DST-NLCs coated with CSO-LA were developed and characterised successfully; NLCs were physically stable and showed improved local drug distribution (which will minimize systemic exposure), delayed drug release, decreased cytotoxicity compared to the drug alone, and less irritancy compared to dutasteride in 70% ethanol. On the 3D reconstructed human epidermis, nanoparticles prepared in water as the continuous phase showed a higher cell viability, indicating that these NLCs were a greater carrier than ethanol based on the possible adverse effects. In addition, the delivery of 5α-reductase inhibitor molecules in the nanocarriers reduced the cytotoxicity of the hair follicle dermal papilla cells. The CSO-LA-coated nanocarriers decreased systemic exposure by restricting drug permeation, as shown by the permeation analysis, where DST-NLCs coated with CSO-LA yielded half of the DST-NLC drug permeation.

Empty NLCs coated with CSO-LA induced cell proliferation, with no irritancy on the 3D reconstructed human epidermis, indicating that this formulation may be used by women for whom dutasteride is not currently indicated. Stearic acid is the key ingredient in the preparation of nanoparticles, and may facilitate cell proliferation along with lauric acid (CSO conjugated with CSO-LA). Stearic and lauric acid are present abundantly in oils extracted from palms and coconuts that are commonly used as traditional hair loss treatments in tropical countries. Hence, the empty formulation of nanocarriers (NLCs) in combination with CSO-LA gave promising results as a hair growth therapy. Based on these findings, the co-delivery of dutasteride in the nanocarriers described in this study with the other hair growth-promoting compounds that generate enhanced clinical results may result in prolonged drug release, minimised cytotoxicity, and potentially synergistic hair proliferation.

## Figures and Tables

**Table 1:** Size distribution, zeta potential, and entrapment efficiency of DST-NLCs before and after coating with 5% CSO-LA at day 1 and day 180 (*n* = 3, mean ± SD).

Parameter	Day 1 at 4–8 °C		Day 180 at 4–8 °C	
	DST-NLCs	DST-NLCs5% CSO-LA	DST-NLCs	DST-NLCs5% CSO-LA
Hydrodynamic diameter (nm)	184.2 ± 2.9	188.4 ± 2.2	265.3 ± 7.8	249.4 ± 7.1
PDI	0.118 ± 0.016	0.124 ± 0.011	0.149 ± 0.015	0.130 ± 0.024
Zeta potential (mV)	−18.0 ± 2.3	+24.8 ± 2.1	−15.8 ± 1.0	+31.4 ± 6.0
Entrapment Efficiency (%)	97.3 ± 1.2%	-	96.6 ± 1.9%	-

**Table 2:** Size distribution and surface charge of Cou-6-NLCs, uncoated and coated with 5% CSO-LA (*n* = 3, mean ± SD).

Formulation	Hydrodynamic Diameter (nm)	PDI	Zeta Potential (mV)
Cou-6-NLCs	192.4 ± 14.4	0.133 ± 0.025	−16.8 ± 0.7
Cou-6-NLCs-5% CSO-LA	217.8 ± 19.4	0.192 ± 0.038	+27.7 ± 0.6

**Table 3:** Dutasteride permeation into the skin after 48 h (*n* = 4, mean ± SD).

Parameter	DST-NLCs	DST-NLCs 5% CSO-LA
Dutasteride in the skin (µg)	4.96 ± 1.36	2.01 ± 0.22
Dutasteride permeated (%)	7.02 ± 1.93	3.45 ± 0.37
Amount of dutasteride in the skin per area, (µg/cm^2^)	6.09 ± 1.09	3.16 ± 0.34
Recovery (%)	93.2 ± 2.1	93.1 ± 1.10

**Table 4:** Comparison of the properties of DST-NLCs, uncoated and coated with CSO-SA and CSO-LA.

Parameter	DST-NLCs	^¥^ DST-NLCs Coated5% CSO-SA	DST-NLCs Coated5% CSO-LA
^a^ Hydrodynamic diameter, nm	184.2 ± 2.9	220.1 ± 11.9	188.4 ± 2.2
^a^ Zeta potential, mV	−18.0 ± 2.3	+26 ± 1.1	+24.8 ± 2.1
^a^ PDI	0.118 ± 0.016	0.149 ± 0.024	0.124 ± 0.011
^a^ Entrapment efficiency, %	97.3 ± 1.2	-	-
^a^ Drug loading, %	3.49 ± 0.10	-	-
^b^ Drug release at 24 h, %	82.6 ± 6.0	58.9 ± 5.9	55.1 ± 11.4
^b^ Skin permeation,%	7.02 ± 1.93	2.91 ± 0.42	3.45 ± 0.37
^b^ MNTC (EC_90_), µM	38.1 ± 13.1	53.6 ± 8.3	39.3 ± 11.5
^a^ Skin irritation	No irritancy	-	No irritancy
Cell uptake	Yes	-	Yes
Skin uptake	Yes	-	Yes

^¥^ Results from [33]. ^a^: *n* = 3, mean ± SD; ^b^: *n* = 4, mean ± SD.
